# Top-down mass spectrometry reveals multiple interactions of an acetylsalicylic acid bearing Zeise’s salt derivative with peptides

**DOI:** 10.1007/s00775-020-01760-9

**Published:** 2020-02-14

**Authors:** Monika Cziferszky, Ronald Gust

**Affiliations:** Department of Pharmaceutical Chemistry, CMBI-Center for Molecular Biosciences, CCB-Centrum for Chemistry and Biomedicine, Innsbruck, Institute of Pharmacy, University of Innsbruck, Innrain 80-82, 6020 Innsbruck, Austria

**Keywords:** Metallodrug, Anticancer drug, Binding site identification, Peptide, Zeise’s salt, Acetylation

## Abstract

Synergistic effects and promising anticancer activities encourage the combination of non-steroidal anti-inflammatory drugs with metallodrugs. Here, we discuss the interactions of an organometallic complex consisting of an acetylsalicylic acid (ASA) moiety attached to a Pt^II^ center via an alkenol linker in a Zeise’s salt-type coordination (ASA–buten–PtCl_3_) with model peptides angiotensin 1 (AT), substance P (Sub P), and ubiquitin (UQ). Top-down mass spectrometry experiments show that the amino acid involved in the initial binding to the metal complex controls the coordination sphere of Pt^II^ in the adducts. The strong *trans* labilizing effect of the coordinating sulfur atom in Met causes fast release of the organic moiety and leads to the formation of dimers and oligomers in the case of Sub P. In contrast, interactions with nitrogen donors in AT result in stable adducts containing the intact ASA–buten–Pt^II^ complex. UQ forms two sets of Pt^II^ adducts, only one of them retains the ASA moiety, which is presumably the result of an unexpected binding geometry. Importantly, UQ is additionally acetylated at various Ser and Lys residues by the ASA–buten–PtCl_3_ complex. Control experiments with ASA are negative. This is the first example of concomitant platination and acetylation of a peptide with an ASA metal complex.

**Electronic supplementary material:**

The online version of this article (10.1007/s00775-020-01760-9) contains supplementary material, which is available to authorized users.

## Introduction

In the quest for novel anticancer agents, the combination of metallodrugs with biologically active compounds is gaining momentum. In particular, metal complexes of non-steroidal anti-inflammatory drugs (NSAIDs) show synergistic effects and promising anticancer activities. A recent review [1] discusses a vast number of metal complexes of NSAIDs and their antibacterial, antifungal, and antiproliferative activities as well as DNA-binding properties, highlighting the advantages and possibilities of these drug candidates. NSAIDs are a group of well-known medications for the treatment of inflammation, pain, and fever, with acetylsalicylic acid (ASA, marketed as Aspirin by Bayer) being a famous example that was already synthesized more than 100 years ago. It exhibits its anti-inflammatory and analgesic effect through acetylation of Ser residues in the active site of cyclooxygenase enzymes (Ser529 in COX-1 and Ser516 in COX-2), which causes irreversible inhibition [2]. All other NSAIDs inhibit COX metabolism through reversible non-covalent attachment to the active site [2]. Today, NSAIDs cover a large number of compounds classified according to their chemical structure, including salicylic acid derivatives, oxicams, sulfonamides, and others.

**Fig. 1:**
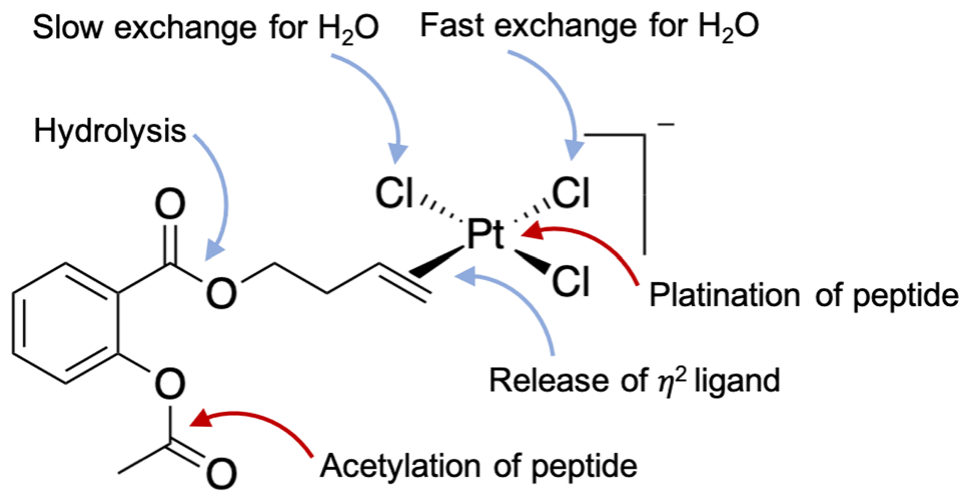
Possible reactions of ASA–buten–PtCl_3_ with peptides in aqueous solutions

Overexpression of COX-2 in different types of tumors, such as prostate, colon, or breast cancer, makes this enzyme an interesting target for the development of novel anticancer drug candidates. Most NSAIDs non-selectively inhibit both COX enzymes. Only a few specific COX-2 inhibitors are on the market. Coordination of NSAIDs to metal centers aims at obtaining antiproliferative activity through a dual mode of action: inhibition of COX and, e.g., ROS generation by the metal. Complexes of Ru^II^, Os^II^, Cu^II^, Re^II^, Fe^II^, Co^II^ and Zn^II^ with indomethacin, naproxen, diclofenac and ibuprofen have been reported [3–11]. Examples of ASA derivatives involve Ir^III^ [12], Pt^IV^ [13], Ag^I^ [14, 15] and various metal carbonyls [16–18]. Most recently, the ASA moiety was linked to Pt^II^ via an alkenol spacer in a Zeise’s salt-type coordination [19], and the influence of the spacer length on the stability and bioactivity of the compounds was determined in our group [20]. In the present study, we chose the most promising candidate ASA–buten–PtCl_3_ (see Fig. 1) to gain an in-depth understanding of the molecular interactions of this type of compound with peptides.

Mass spectrometry (MS) is a powerful analytical tool to study the adduct formation of metallodrugs with biomolecules on account of its sensitivity and ability to cope with complex mixtures. The possibility to isolate any gaseous ion of interest, including low abundant species, and dissociate them via an array of different fragmentation techniques (MS/MS) enables in-depth structural analysis of these adducts. Ion dissociation techniques are either based on collisions (collision-induced dissociation CID, higher-energy collisional dissociation HCD) or the transfer/capture of electrons (electron capture dissociation ECD, electron transfer dissociation ETD, and electron detachment dissociation EDD) or the absorption of photons (infrared multiphoton dissociation IRMPD, blackbody infrared radiative dissociation BIRD, and ultraviolet photodissociation UVPD). In the context of metallodrug research, ETD has been reported to perform better than CID and HCD for Pt^II^ peptide adducts [21], while ECD proved to be challenging for Ir^III^ peptide adducts due to electron quenching, yet it was successfully used to determine the modification sites [22]. Photodissociation methods have shown promising results for pinpointing cisplatin-binding sites on oligonucleotides [23]. Various MS-based approaches including high-resolution top-down MS, bottom-up methods, and ion-mobility MS are pursued to obtain information about novel metal compounds in a biological and medicinal setting, and ultimately to understand their mode of action [24, 25]. Top-down methodologies are particularly interesting, as little sample preparation is required and, consequently, the danger of further alterations of the newly formed metal–biomolecule bond is minimized. The unambiguous determination of metallation sites for Pt^II^, Ru^II^, Ir^III^, and Os^II^ compounds on various peptides [26–28], proteins [29, 30], and oligonucleotides [31–33] was achieved through top-down MS.

Cisplatin is a potent anticancer drug, which exerts its mode of action through binding to two adjacent N7 in guanine residues of DNA [34]. However, up to 98% of cisplatin in the blood plasma is protein-bound within 1 day of injection [35]. While protein binding may serve as transport mechanism, the high reactivity of Pt^II^ complexes with enzymes is likely a primary cause for the large number of dose-limiting side effects of Pt^II^-based chemotherapy [36]. Sulfur-containing Met and Cys as well as the nitrogen donor His are generally accepted binding partners for cisplatin and other Pt^II^ compounds [37–43]. While thiols and thioethers rapidly displace chlorido ligands on cisplatin directly, reactions with amines require a rate-determining aquation step first [44].

In the current study, we seek to get a better understanding of the molecular interactions of ASA–buten–PtCl_3_ with three different model peptides as reaction partners. Angiotensin I (AT) is a ten-amino acid peptide hormone containing two His residues as possible metallation sites. Substance P (Sub P), an eleven-amino acid neurotransmitter, contains a C-terminal Met amide. And ubiquitin (UQ) consists of 76 amino acids with an N-terminal Met and one His at position 68. The sequences of the three peptides are depicted in Fig. 3. An MS-based study on the reaction products of Zeise’s salt with AT and UQ was published recently by our group [45], where we demonstrated that *trans* labilizing effects play a crucial role in the overall peptide metallation by Pt^II^ complexes. Replacement of the ethylene ligand in Zeise’s salt with an ASA–buten moiety led to remarkable COX inhibition (three times higher than ASA alone) and antiproliferative activity (IC_50_ ~ 30 µM in HT-29 and MCF-7 cell lines) [20]. Here, we shine light on the underlying molecular interactions. A particularly interesting question is, if this type of ASA derivative retains its potential to acetylate susceptible amino acids. Few studies have addressed this question so far. Hey-Hawkins et al*.* reported acetylation of COX-1 and COX-2 at various peripheral Lys and Ser residues by asborin, a carbaborane with ASA-like features. However, no acetylation was found in the active sites of both COX enzymes [46]. Similarly, a Co^0^-carbonyl ASA complex of our group did not acetylate Ser516 in COX-2 but rather acetylated a number of Lys residues [18]. Another example for the successful combination of an alkylating agent with a metal compound is the chlorambucil-functionalized Ru^II^ complex of Nazarov et al*.* [47] designed to crosslink DNA with proteins by interactions of the metal with amino acids and simultaneous alkylation of N7 in guanine.

## Experimental

### Chemicals and reagents

Angiotensin I (human, acetate salt hydrate, > 90%), ubiquitin (from bovine erythrocytes, > 98%), and substance P (acetate salt hydrate, > 95%) were obtained from Sigma-Aldrich and used as received. Solvents were purchased in MS quality from Sigma-Aldrich. Synthesis, stability and biological data of ASA–buten–PtCl_3_ are reported elsewhere [20].

### Incubation

Peptides (10 µM) were incubated with ASA–buten–PtCl_3_ in 1:5 ratio in pure water at 37 °C. Aliquots were taken after 5 min, 2 h, 24 h, and 48 h, diluted with 0.1% formic acid in acetonitrile, and directly infused into the mass spectrometer as described below. A final measurement was performed after 7 days of incubation. In the case of UQ, samples were centrifuged on prewashed nanosep centrifugal devices with a molecular weight cut-off of 3500 Da at 8500 rpm and washed with water twice to remove any excess metal complex. Finally, the remaining peptide was diluted as described above and measured. All aqueous peptide solutions with ASA–buten–PtCl_3_ or ASA were at pH 6, which corresponds roughly to the isoelectric point of UQ. The pH did not change over the course of the reaction.

### ESI–MS analysis

Samples were measured on an Orbitrap Elite (Thermo Fisher Scientific) in positive mode under standard operating conditions using the HESI source (heated electrospray ionization) and the syringe pump. HCD experiments were performed manually on all ions of interest with an isolation window of 3–7 *m*/*z*. The normalized collision energy (NCE) was increased stepwise. Data were analyzed using Xcalibur software and the Apm^2^s software tool developed by Dyson et al*.* [43] (https://ms.cheminfo.org/apm2s/index.html). Search parameters were as follows: common zone was set to “second”, zone low − 2.5, high 4.5, maximal length of internal fragments was set to 10, and neutral loss was only ticked in the case of Sub P.

## Results and discussion

The *trans* labilizing effect of all relevant ligands in this study increases in the order H_2_O < NR_3_ < Cl^−^ < SR_2_ < CH_2_ = CHR [48]. In aqueous solution, the *trans* effect of ethylene in Zeise’s salt causes aquation of the *trans* position in less than 2 min [49].

The stability of ASA–buten–PtCl_3_ was assessed in pure water and 0.9% NaCl by capillary electrophoresis as reported previously [20]. While slow ester cleavages were observed (*τ*_½_ = 69.6 ± 3.0 h), no redox reactions, as is the case for Zeise’s salt in H_2_O [50], were found. The details of the aquation of ASA–buten–PtCl_3_ remain elusive at this point due to inherent changes in the charge state of the Pt^II^ complex that pose a challenge for an observation by mass spectrometry. Initially, [PtCl_3_(C_13_H_14_O_4_)]^−^ was observed at *m*/*z* 535.0 in the negative mode, while the first aquation product PtCl_2_(C_13_H_14_O_4_)(H_2_O) is neutral and could not be detected. After incubation for 72 h in water, the appearance of [PtCl(C_13_H_14_O_4_)(H_2_O)_2_]^+^ at *m*/*z* 500.0 showed that aquation happens at a relevant time scale.

**Fig. 2:**
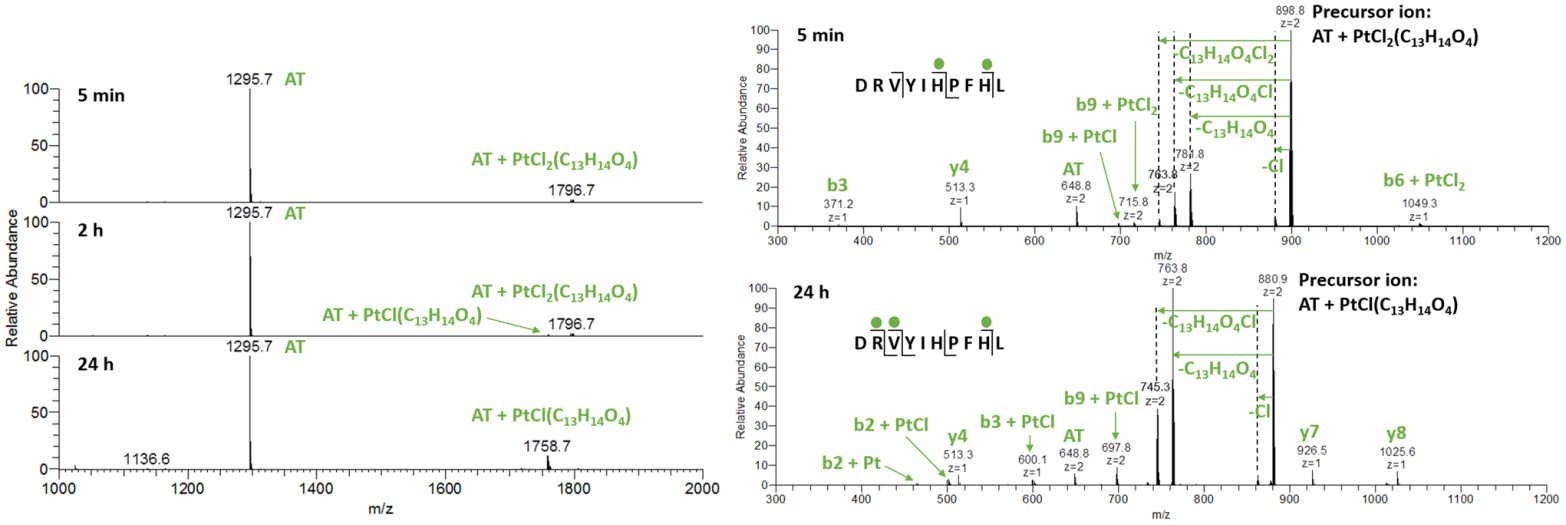
Left: deconvoluted mass spectra of AT after incubation with ASA–buten–PtCl_3_ at different points in time. Right: HCD fragmentation spectra at 15% NCE of the AT adducts after 5 min (top) and 24 h (bottom)

**Fig. 3:**
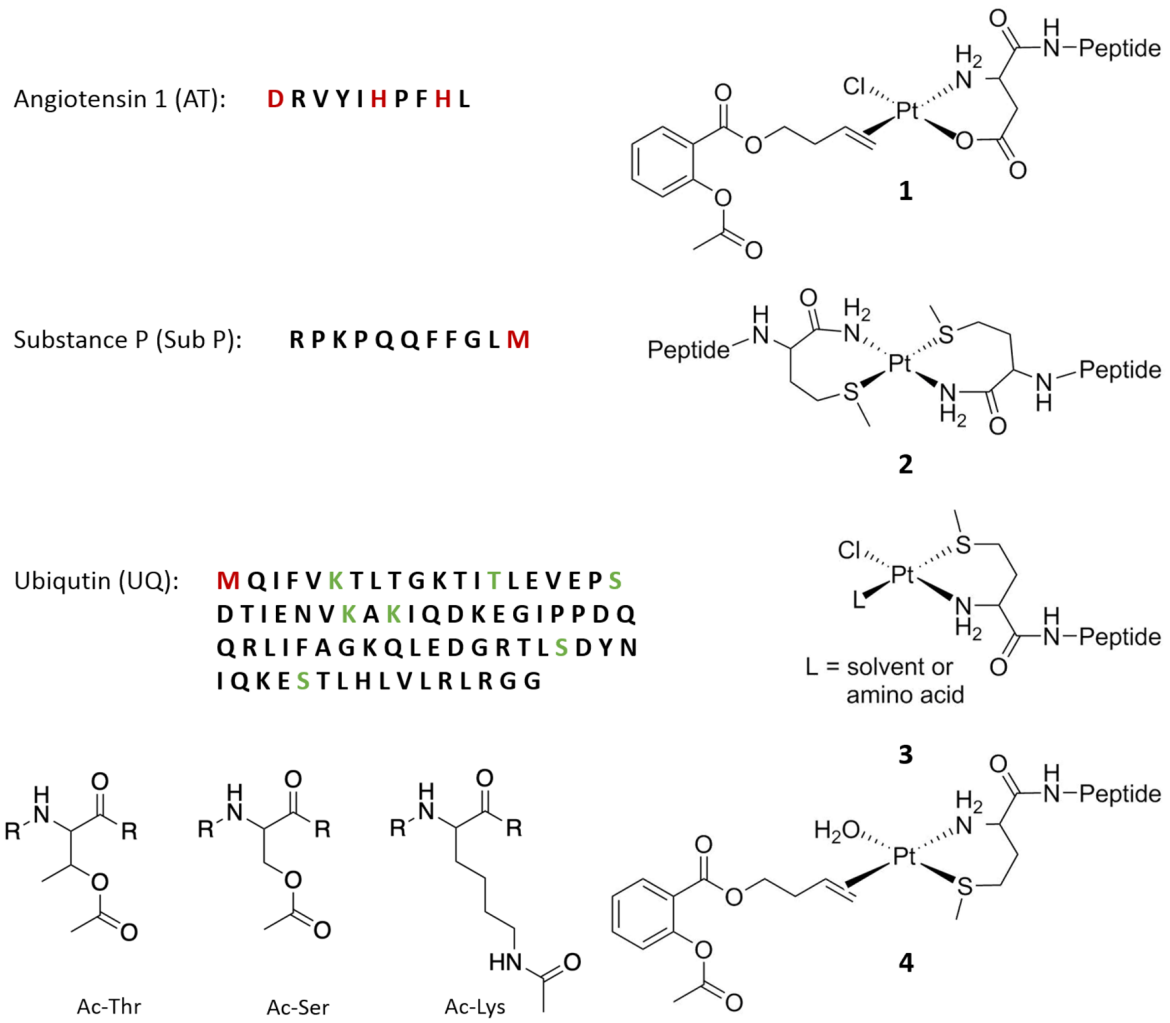
Sequences of the peptides used in this study with platination sites marked in red and acetylation sites marked in green. Proposed structures (without charges) for the adducts formed upon incubation with ASA–buten–PtCl_3_ are on the right

When AT was incubated with ASA–buten–PtCl_3_, the first adduct observed was AT + PtCl_2_(C_13_H_14_O_4_) with *m*/*z* 1796.7 (see Fig. 2), where one chlorido ligand has been replaced by a suitable donor on the peptide. Presumably, the chlorido ligand *trans* to the olefin has been exchanged for an aqua ligand, which in turn enabled replacement by a nitrogen of His. After 2 h of incubation, a second signal with *m*/*z* 1758.7 was detected, which corresponds to AT + PtCl(C_13_H_14_O_4_). After 24 h, this adduct reaches roughly 15% relative abundance and no further changes were observed in the following 6 days. The ASA–buten moiety remained attached to Pt^II^, and no ester bond cleavages were found. Most likely, a stable bidentate coordination between the biomolecule and the Pt^II^ center was formed (see structure **1** in Fig. 3).

Throughout this study, fragmentation experiments were performed in the form of HCD. The fragmentation energy was increased stepwise to monitor both, the first-bond cleavages (typically 10–15% NCE) and a maximized number of platinated fragments with higher energies (typically 25–30% NCE). Data were analyzed with Xcalibur (Thermo) and Apm^2^s software tool that enables determination of internal metallated fragments on top of the usual N-terminal and C-terminal fragments [43].

HCD fragmentations of the species at *m*/*z* 1796.7 with 15% NCE indicated His6 as first point of attachment for the metal complex. Low fragmentation energy resulted in the loss of the ASA–buten moiety; however, the newly formed bond to Pt^II^ was strong enough for a number of platinated fragments to be registered (see Fig. 2). The ions b6 + PtCl_2_ and the corresponding y4 suggest that the platinated AT breaks next to the Pt^II^ modification site at His6. However, platination at His9 cannot be excluded. The appearance of a non-platinated b3 fragment is noteworthy, since the picture changes over time. After 24 h, HCD fragmentation with 15% NCE of the species with *m*/*z* 1758.7 resulted in N-terminal-platinated fragments (b2/3 + Pt(Cl)_0–1_) and corresponding non-platinated y7 and y8 fragments (see Fig. 2). This finding indicates an internal rearrangement of the PtCl(C_13_H_14_O_4_) moiety within 24 h towards the N-terminus, where a bidentate complexation to Asp can easily be accomplished, as shown in Fig. 3, structure **1**. Fragmentation experiments with 30% NCE produced a number of platinated fragments confirming the N-terminus and both His residues as binding sites (see supplementary material, Table S1).

**Fig. 4:**
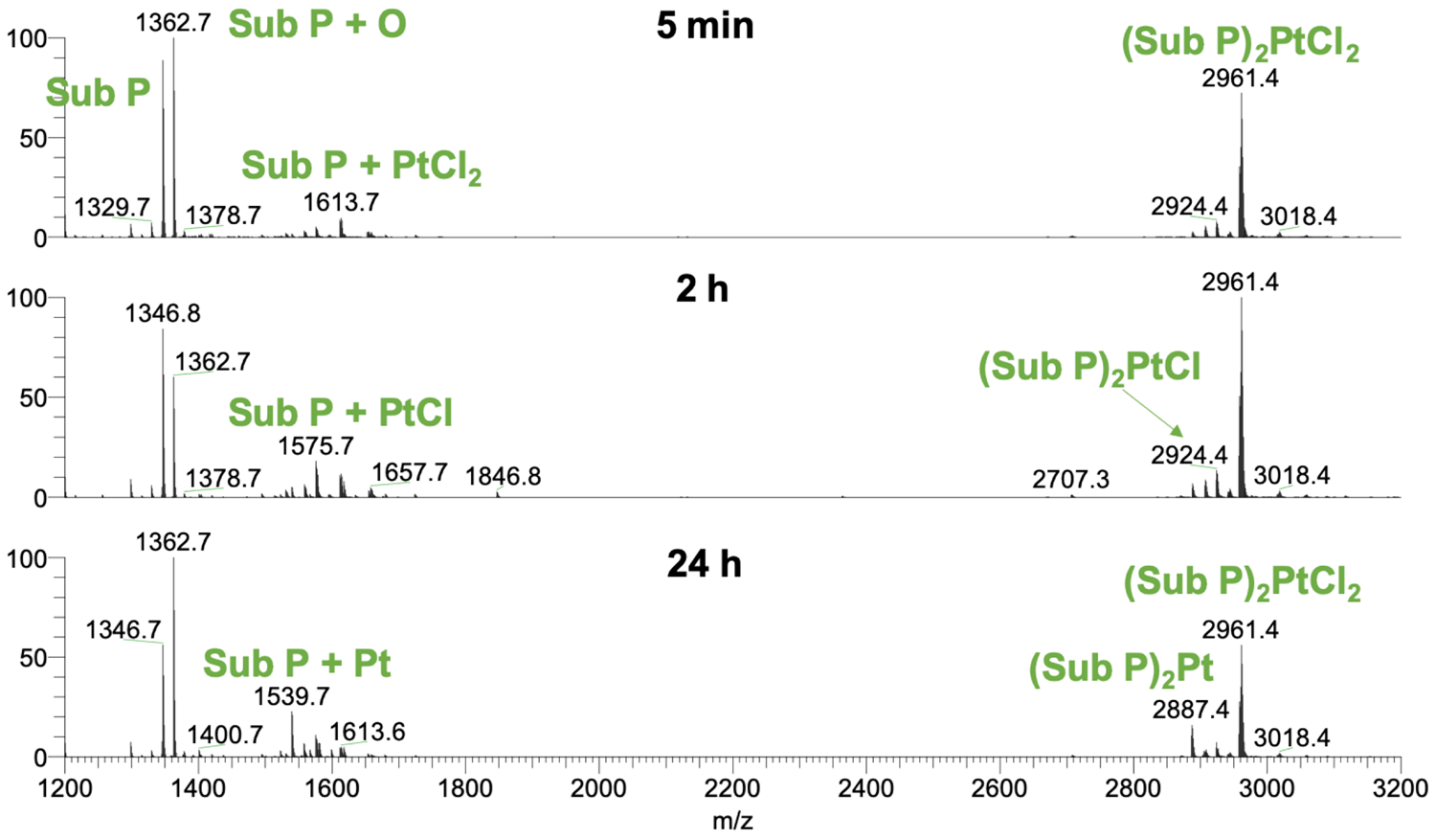
Deconvoluted mass spectra of Sub P after incubation with ASA–buten–PtCl_3_

Experiments with Sub P resulted in a distinctly different picture. Sub P contains one Met amide residue on the C-terminus. This offers the possibility to form a bidentate coordination to Pt^II^ via sulfur and either the C-terminal amide nitrogen or the first backbone nitrogen resulting in a 6- or 7-membered ring, respectively. The measurement after 5 min of incubation showed a high abundant adduct with *m*/*z* 2961.4 (see Fig. 4) that corresponds to the dimer (Sub P)_2_PtCl_2_. This finding can be rationalized by a substitution of the *trans* chlorido ligand by the Met11 thioether. Sulfur has a strong *trans* labilizing effect and, consequently, the bond to the olefin is weakened upon coordination. Another entity of Sub P is able to bind to the Pt^II^ center, while the organic moiety is released. The two chlorido ligands remain attached at first, but some release is observed over time. The species with *m*/*z* 2887.4 resembles a Sub P dimer that is crosslinked by Pt^II^ and presumably looks like compound **2** in Fig. 3.

**Fig. 5:**
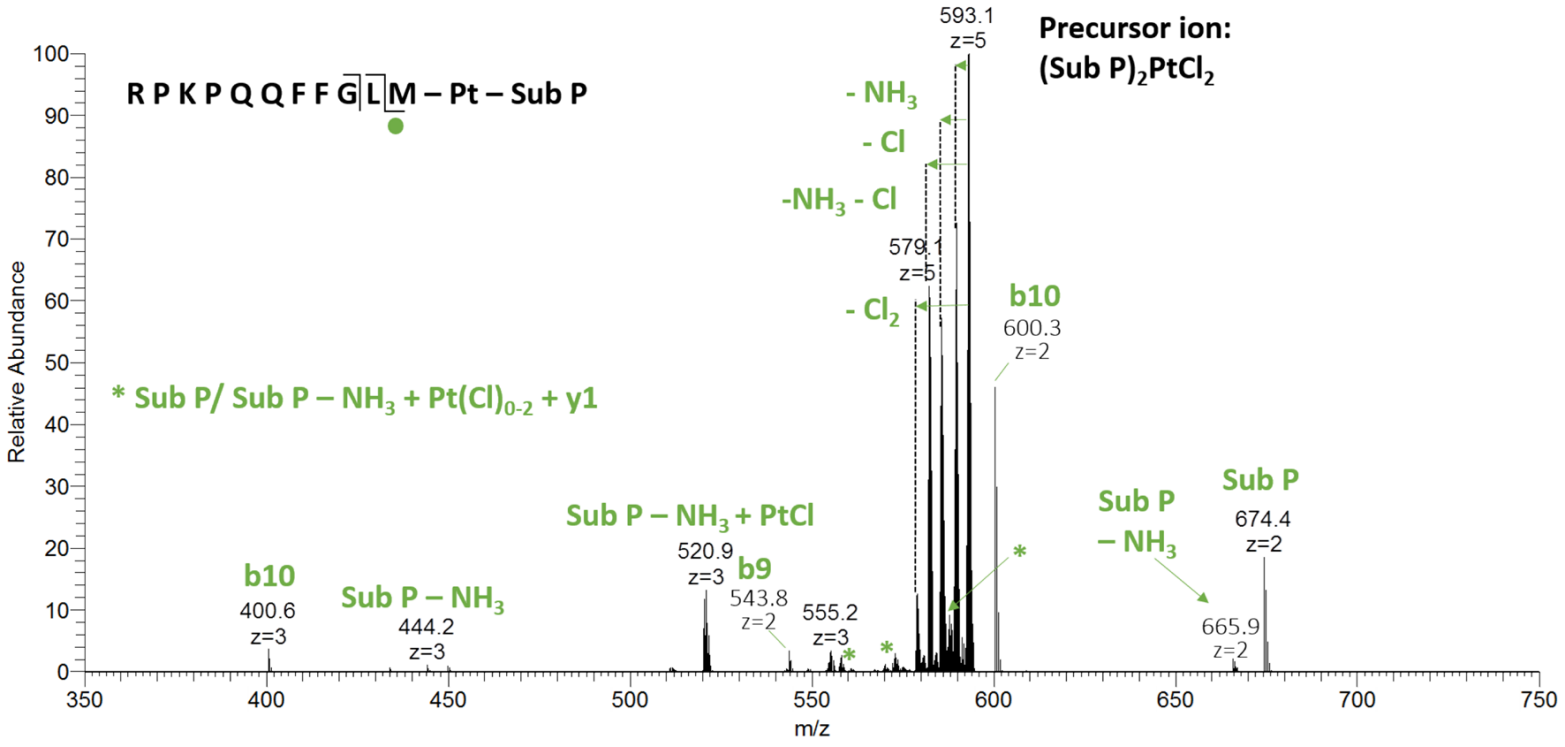
HCD fragmentation spectrum at 10% NCE of the dimer (Sub P)_2_PtCl_2_

HCD experiments of the dimer (Sub P)_2_PtCl_2_ at 15% NCE resulted mainly in the loss of chlorido ligands and NH_3_ from side chains (see Fig. 5). The formation of free Sub P at *m*/*z* 674.4 and Sub P − NH_3_ at *m*/*z* 665.9 and *m*/*z* 444.2, in charge states 2 and 3, respectively, confirms the dissociation of the dimer. Interestingly, b10 can be seen in comparatively high abundance, which enabled the assignment of some corresponding fragments that still contain the Pt^II^ crosslink. These are the triply charged species Sub P + PtCl_2_ + y1 at *m*/*z* 587.9, Sub P − NH_3_ + PtCl + y1 at *m*/*z* 570.2 and Sub P − NH_3_ + Pt + y1 (see supplementary material, Figure S2 for isotopic distributions). Higher fragmentation energies primarily led to the loss of the Pt^II^ moiety and no small platinated fragments could be detected. However, the positioning of Pt^II^ on Met is quite certain based on the data observed and in accordance with HSAB theory. A full list of fragments can be found in the supplementary material (Table S2).

Higher oligomers up to a tetramer of Sub P were observed at low abundance (see Table 1). Also, the MS signal was in general much worse than in the experiments with AT and declined over time. These observations point to Pt^II^-induced peptide aggregation and crosslinking. For comparison, Sub P was incubated with ASA–buten–PtCl_3_ at equimolar ratio (see supplementary material, Fig. S1). In this case, the signal-to-noise ratio was better. The main adduct observed was Sub P + PtCl_2_, which lost its chlorido ligands over time. The Pt^II^-coordinated Sub P dimer appeared at significantly lower abundance, and no higher oligomers were observed. Similar findings were reported by Merlino et al*.* [51], who investigated the formation of cisplatin interprotein crosslinks and platinated oligomers of RNase A.

**Table 1:** Adducts and oligomers of Sub P after 48 h incubation with ASA–buten–PtCl_3_

Species	*m*_exp_	*m*_calc_	Error (ppm)
Sub P	1346.7311	1346.7276	− 2.60
Sub P + Pt	1539.6807	1539.6825	1.17
Sub P + PtCl	1575.6577	1575.6591	0.89
Sub P + PtCl_2_	1613.6343	1613.6379	2.23
(Sub P)_2_Pt	2887.4143	2887.4131	− 0.42
(Sub P)_2_PtCl	2924.3925	2924.3910	− 0.51
(Sub P)_2_PtCl_2_	2961.3670	2961.3680	0.34
(Sub P)_3_Pt_2_Cl_2_	4501.0494	4501.0387	− 2.38
(Sub P)_3_Pt_2_Cl_4_	4575.0009	4574.9988	− 0.46
(Sub P)_4_Pt_2_Cl_3_	5886.7444	5886.7533	1.51
(Sub P)_4_Pt_3_Cl_6_	6188.6440	6188.6297	− 2.31

Neither AT nor Sub P were acetylated by the ASA moiety.

UQ is the largest peptide in this study with 76 amino acids, a Met residue at the N-terminus, His68, and some weaker binding partners (free –OH and –COOH residues) for Pt^II^ that have been identified before [43].

**Fig. 6:**
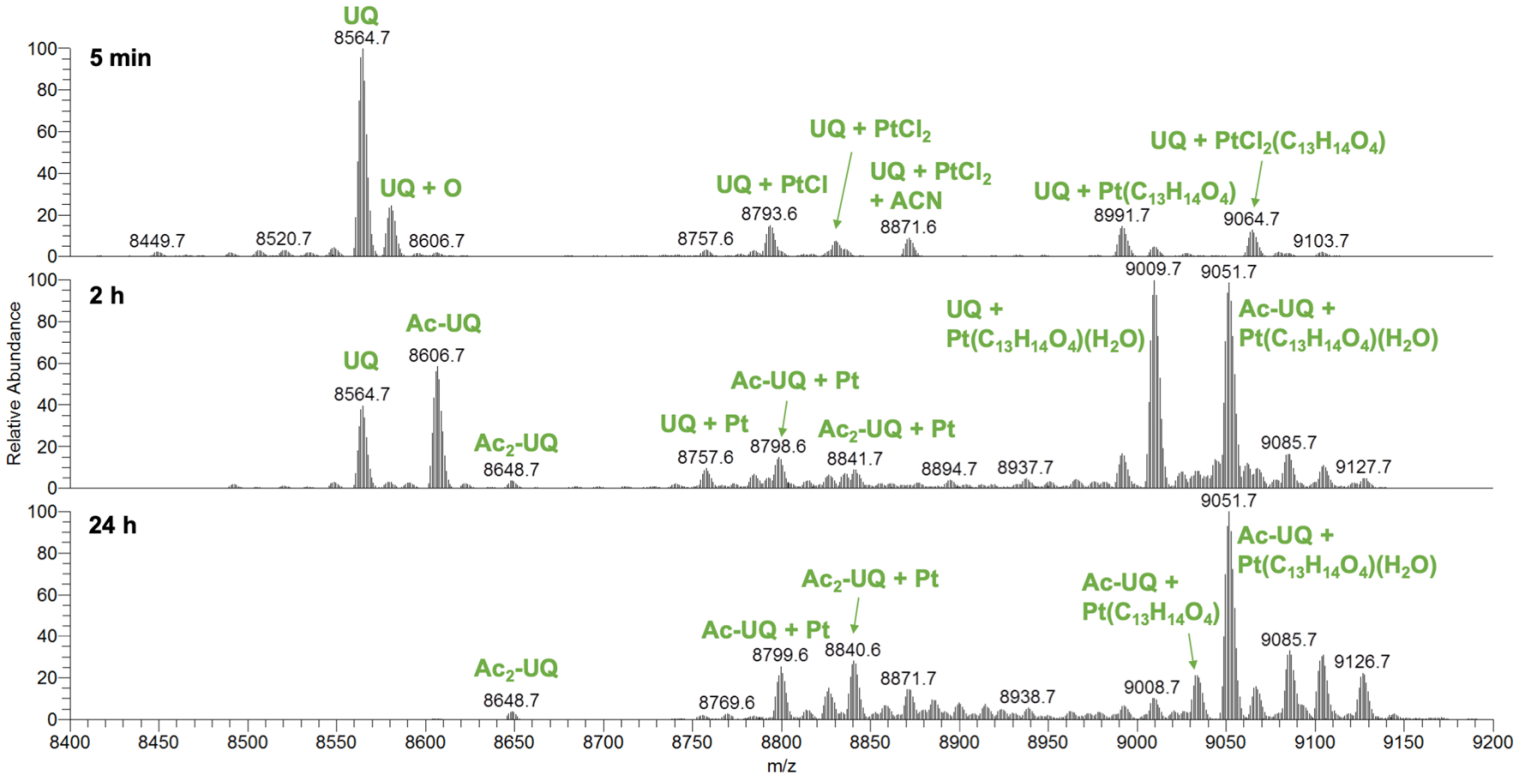
Deconvoluted mass spectra of UQ after incubation with ASA–buten–PtCl_3_

Upon incubation with ASA–buten–PtCl_3_, two sets of adducts appeared after 5 min, one with the PtCl_0–2_(C_13_H_14_O_4_) moiety attached and the other with PtCl_0–2_ only (see Fig. 6). The latter can be explained by an attack of the Met sulfur *trans* to the olefin and subsequent release of the organic ligand (see structure **3** in Fig. 3). The binding site of Pt^II^ is confirmed by the appearance of small Pt^II^ containing fragments a3 + Pt (*m*/*z* 540.2) and b4 + Pt (*m*/*z* 713.2).

**Fig. 7:**
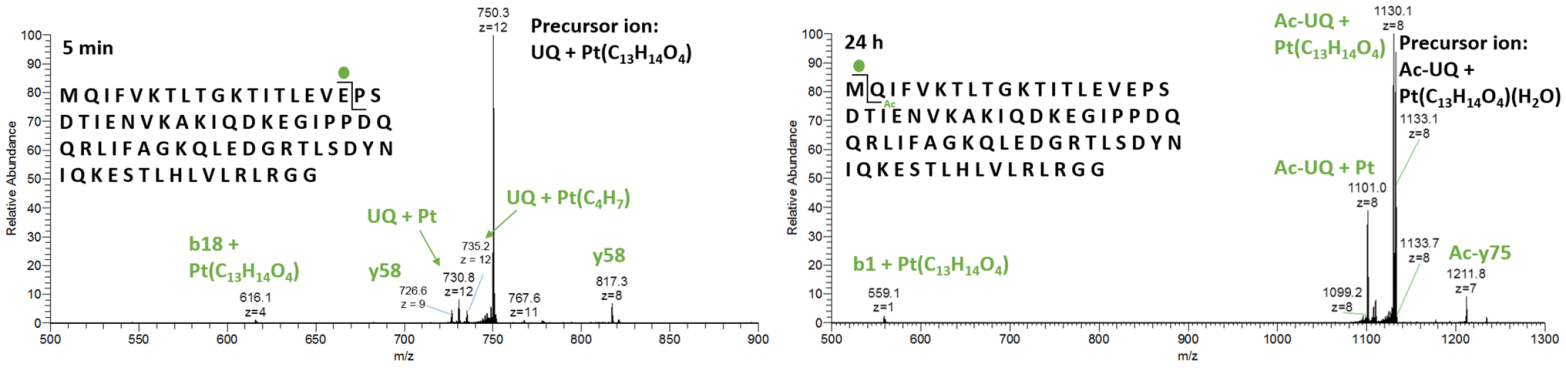
HCD fragmentation spectra at 15% NCE of UQ adducts

As observed for both UQ and Sub P, the *trans* labilizing effect of the thioether in Met caused fast release of the organic moiety. Hence, the retention of the ASA–buten moiety in the second set of signals can only be rationalized by coordination of a nitrogen (or less likely oxygen) ligand* trans *to the olefin. HCD fragmentation of the precursor *m*/*z* 750.3 (*z* = 12, UQ + Pt(C_13_H_14_O_4_)) with low energy shows two corresponding ions b18 + Pt(C_13_H_14_O_4_) and y58 (see Fig. 7). Besides the N-terminal Met, there are several nitrogen and oxygen donors in the b18 fragment that may serve as binding partners; however, further fragmentations with 25% NCE point to the N-terminal Met as binding site. Fragments a3/4 + Pt and b3/4 + Pt were detected (see supplementary material, Table S4 for a full list of fragments). One possible explanation for the retention of the ASA–buten moiety in this case is the formation of isomer **4** depicted in Fig. 3, where the N-terminal nitrogen is in the *trans* position to the olefin and sulfur is *cis.* After 24 h of incubation, HCD fragmentation of the precursor *m*/*z* 1133.1 [*z* = 8, Ac-UQ + Pt(C_13_H_14_O_4_)(H_2_O), see below] yielded the interesting fragment b1 + Pt(C_13_H_14_O_4_) and the corresponding acetylated y75 fragment (see Fig. 7). This is further evidence for the formation of isomer **4**.

Importantly, UQ was acetylated by ASA–buten–PtCl_3_ as indicated by a high abundant signal for Ac-UQ with *m*/*z* 8606.7 and Ac_2_-UQ with *m*/*z* 8648.7 after 2 h. These signals have almost disappeared after 24 h, and instead, UQ is found to be both, acetylated and platinated (see Fig. 6). To the best of our knowledge, this is the first example of two different chemical modifications on a single peptide by an NSAID metal complex. After 24 h, Ac-UQ + Pt(C_13_H_14_O_4_)(H_2_O) is the most abundant species and no further changes were observed in the next 6 days. Control experiments with ASA did not show any acetylations (see supplementary material, Fig. S3). HCD fragmentations revealed Lys6, Thr7, Thr14, Ser20, Thr22, Lys27, Lys29, Thr55, Ser57, Ser65, and Thr66 as possible acetylation sites (see Table 2 and Fig. 3). Clearly acetylation happened in a non-specific fashion.

**Table 2:** Acetylated fragments of UQ smaller than ten amino acids; precursor ion *m*/*z* 1133.1 [*z* = 8, Ac-UQ + Pt(C_13_H_14_O_4_)(H_2_O)]

Acetylated fragment	Amino acids	*m*_calc_	*m*_exp_	Similarity	Error (ppm)
a20y58	Pro-Ser(Ac)	199.1083	199.1077	88.4	2.39
a29y49	Ala-Lys(Ac)	214.1556	214.1550	76.1	2.30
a28y50	(Ac)Lys-Ala	214.1556	214.1550	76.1	2.30
a57y21	Leu-Ser(Ac)	215.1396	215.1390	74.4	1.87
b29y49	Ala-Lys(Ac)	242.1505	242.1499	78.5	2.92
b28y50	(Ac)Lys-Ala	242.1505	242.1499	78.5	2.92
b57y21	Leu-Ser(Ac)	243.1345	243.1339	78.9	1.97
b27y51, b6y72	Val-Lys(Ac)	270.1818	270.1812	76.7	2.21
a67y12	Ser-Thr-Leu^a^	316.1872	316.1867	88.0	2.67
a57y22	Thr-Leu-Ser^a^	316.1872	316.1867	88.0	2.67
b67y12	Ser-Thr-Leu^a^	344.1822	344.1816	88.0	2.48
b57y22	Thr-Leu-Ser^a^	344.1822	344.1816	88.0	2.48
b58y21	Leu-Ser(Ac)-Asp	358.1614	358.1609	79.2	2.78
b7y72	Val-Lys-Thr^a^	371.2294	371.2289	81.7	2.31
b6y73	Phe-Val-Lys(Ac)	417.2502	417.2496	85.0	2.39
a17y63	Thr(Ac)-Leu-Glu-Val	457.2662	457.2657	75.5	2.55
b31y49	Ala-Lys(Ac)-Ile-Gln	483.2931	483.2926	72.5	3.22
a22y60	Val-Glu-Pro-Ser-Asp-Thr^a^	643.2939	643.2933	79.4	2.80
b6	Met-Gln-Ile-Phe-Val-Lys(Ac)	789.4333	789.4328	78.1	3.11

^a^Two possible acetylation sites in the sequence

## Conclusions

Molecular interactions of the NSAID metal complex ASA–buten–PtCl_3_ with three model peptides were investigated by top-down mass spectrometry. The Pt^II^ complex formed different adducts depending on the amino acids available for binding. *Trans* labilizing effects played a crucial role in the outcome of the reaction. Depending on the type of ligand that coordinated *trans* to the olefin, the organic moiety was released quickly or retained for the duration of the experiment, i.e. 7 days. Based on MS results, we conclude that within 24 h, Pt^II^ is coordinated in a bidentate fashion in all cases. Dimer formation and oligomerization were observed in the case of Sub P. Most importantly, we could prove that the combination of an NSAID with a metal complex truly led to a compound that can add two different chemical modifications to a peptide at the same time. Ubiquitin was found to be both acetylated and platinated by ASA–buten–PtCl_3_. Control experiments with ASA did not show any acetylation.

Increasing our general understanding of the interactions between metal complexes and biomolecules is pivotal in the development of better metallodrug candidates.

## Electronic supplementary material

Below is the link to the electronic supplementary material.

Supplementary file1 (PDF 425 kb)
