# Supplementary material for: Top-down mass spectrometry reveals multiple interactions of an acetylsalicylic acid bearing Zeise’s salt derivative with peptides
Source: J Biol Inorg Chem. 2020 Feb 14;25(2):285–93. doi: 10.1007/s00775-020-01760-9 (PMC7082381; doi:10.1007/s00775-020-01760-9)
Supplement: Supplementary file 1 — Supplementary file1 (PDF 425 kb) [file 775_2020_1760_MOESM1_ESM.pdf]

**Top-down Mass Spectrometry Reveals Multiple Interactions of an Acetylsalicylic Acid Bearing Zeise's Salt Derivative with Peptides**

Monika Cziferszky, Ronald Gust*

*Department of Pharmaceutical Chemistry, Institute of Pharmacy, University of Innsbruck, CMBI - Center for Molecular Biosciences Innsbruck, University of Innsbruck, CCB - Centrum for Chemistry and Biomedicine, Innrain 80-82, A-6020 Innsbruck, Austria*

*email:* ronald.gust@uibk.ac.at

## Angiotensin 1

*Table S1: Full list of fragments obtained from HCD fragmentation at 30% NCE of the precursor m/z 880.9 (z =2) AT + PtCl($C_{13}H_{14}O_4$) after 24 h (* indicates fragments that were assigned manually).*

| fragment | $m_{calc.}$ | $m_{exp.}$ | Similarity | Charge | error [ppm] |
|---|---|---|---|---|---|
| His - CO | 110.0718 | 110.0713 | 59.2 | 1 | 1.02 |
| Arg - $NH_3$ | 112.0875 | 112.0869 | 93.0 | 1 | 1.02 |
| y1 | 132.1025 | 132.1019 | 89.9 | 1 | 0.90 |
| Tyr | 136.0762 | 136.0757 | 93.2 | 1 | 1.80 |
| His | 138.0667 | 138.0662 | 60.5 | 1 | 1.87 |
| Arg - $NH_3$ | 140.0824 | 140.0818 | 92.4 | 1 | 1.63 |
| a8y4 | 217.1341 | 217.1335 | 91.9 | 1 | 2.01 |
| a6y6 | 223.1559 | 223.1553 | 69.0 | 1 | 2.12 |
| b7y5 | 235.1195 | 235.1190 | 82.8 | 1 | 2.29 |
| a4y8 | 235.1447 | 235.1441 | 76.9 | 1 | 2.11 |
| b8y4 | 245.1290 | 245.1285 | 57.8 | 1 | 2.32 |
| b6y6 | 251.1508 | 251.1503 | 76.6 | 1 | 2.27 |
| b2 - $NH_3$ | 255.1093 | 255.1088 | 88.6 | 1 | 2.30 |
| b4y8 | 263.1396 | 263.1390 | 81.3 | 1 | 2.27 |
| y2 | 269.1614 | 269.1608 | 88.7 | 1 | 2.37 |
| b2 | 272.1359 | 272.1353 | 72.4 | 1 | 2.41 |
| b5y7 | 277.1552 | 277.1547 | 83.7 | 1 | 2.21 |
| b9y3 | 285.1352 | 285.1346 | 91.8 | 1 | 2.34 |
| a3 | 343.2094 | 343.2088 | 83.4 | 1 | 2.40 |
| b3 - $NH_3$ | 354.1777 | 354.1772 | 82.2 | 1 | 2.25 |
| a9y4 | 354.1930 | 354.1925 | 77.6 | 1 | 2.28 |
| a8y5 | 354.1930 | 354.1925 | 77.6 | 1 | 2.28 |
| b3 | 371.2043 | 371.2037 | 83.6 | 1 | 2.42 |
| b9y4 | 382.1879 | 382.1874 | 83.2 | 1 | 2.29 |
| b8y5 | 382.1879 | 382.1874 | 83.2 | 1 | 2.29 |
| b6 | 785.4184 | 392.7087 | 86.0 | 2 | 2.22 |
| a2 - $NH_3$ - $H_2O$ + Pt | 404.0686 | 404.0681 | 67.3 | 1 | 2.54 |
| b6y7 | 414.2141 | 414.2136 | 91.5 | 1 | 2.34 |
| y3 | 416.2298 | 416.2292 | 83.2 | 1 | 2.40 |
| a2 - $H_2O$ + Pt | 421.0952 | 421.0946 | 80.8 | 1 | 2.41 |
| a2 + Pt | 437.0901 | 437.0907 | 92.4 | 1 | -0.05 |
| b7 | 882.4712 | 441.2350 | 82.4 | 2 | 2.30 |
| b2 + Pt | 465.0850 | 465.0856 | 90.6 | 1 | 0.20 |
| a2 + PtCl | 473.0662 | 473.0674 | * | 1 | 2.54 |

| | | | | | |
|---|---|---|---|---|---|
| a6y8 | 485.2876 | 485.2871 | 84.1 | 1 | 2.49 |
| a6 + PtCl | 986.3493 | 493.1741 | 80.6 | 2 | 2.92 |
| b8y6 | 495.2720 | 495.2714 | 82.4 | 1 | 2.51 |
| a8 | 1001.5447 | 500.7718 | 84.2 | 2 | 2.40 |
| b2 + PtCl | 501.0612 | 501.0626 | * | 1 | 2.79 |
| y8 | 1026.5651 | 513.2820 | 64.6 | 2 | 2.39 |
| a3 - $NH_3$ + Pt | 519.1320 | 519.1325 | 81.8 | 1 | 0.70 |
| b9y5 | 519.2468 | 519.2463 | 81.2 | 1 | 2.47 |
| b4 | 534.2676 | 534.2671 | 84.2 | 1 | 2.53 |
| a3 + Pt | 536.1585 | 536.1591 | 91.5 | 1 | 0.53 |
| a8y5 + Pt | 547.1421 | 547.1427 | 84.9 | 1 | 0.79 |
| a9y4 + Pt | 547.1421 | 547.1427 | 84.9 | 1 | 0.79 |
| a9 | 1138.6036 | 569.3013 | 84.5 | 2 | 2.44 |
| a3 + PtCl | 572.1347 | 572.1362 | * | 1 | 2.62 |
| b9 | 1166.5985 | 583.2987 | 85.9 | 2 | 2.57 |
| y9 | 1182.6662 | 591.3326 | 85.1 | 2 | 2.86 |
| b3 + PtCl | 600.1296 | 600.1314 | * | 1 | 3.00 |
| b7y8 | 610.3353 | 610.3348 | 86.1 | 1 | 2.37 |
| b8 + Pt | 1224.5044 | 612.2516 | 73.9 | 2 | 4.84 |
| a8 + PtCl | 1230.4705 | 615.2347 | 82.5 | 2 | 2.43 |
| a5 | 619.3568 | 619.3562 | 85.7 | 1 | 2.36 |
| a9y9 + PtCl | 1252.5025 | 626.2507 | 85.4 | 2 | 2.67 |
| b9y6 | 632.3309 | 632.3303 | 86.0 | 1 | 2.38 |
| AT - $H_2O$ | 1279.6826 | 639.8407 | 78.9 | 2 | 2.78 |
| b9y9 + PtCl | 1280.4974 | 640.2481 | 87.5 | 2 | 2.63 |
| AT | 1297.6931 | 648.8460 | 84.3 | 2 | 2.61 |
| y5 | 650.3415 | 650.3409 | 88.8 | 1 | 2.51 |
| a9 - $H_2O$ + Pt | 1315.5578 | 657.7784 | 66.4 | 2 | 2.61 |
| b8y7 | 658.3353 | 658.3348 | 86.8 | 1 | 2.61 |
| b6y9 | 669.3837 | 669.3831 | 87.1 | 1 | 2.58 |
| a9 + PtCl | 1367.5294 | 683.7642 | 87.2 | 2 | 2.93 |
| b9 + PtCl | 1395.5243 | 697.7616 | 89.4 | 2 | 3.06 |
| a4 + Pt | 699.2218 | 699.2224 | 86.3 | 1 | 0.95 |
| b4 + Pt | 727.2168 | 727.2173 | 87.0 | 1 | 1.40 |
| AT - $H_2O$ + Pt | 1472.6317 | 736.3153 | 72.9 | 2 | 2.77 |
| AT - $NH_3$ + Pt | 1473.6157 | 736.8073 | 70.2 | 2 | 2.82 |
| AT + Pt | 1490.6423 | 745.3206 | 92.2 | 2 | 2.89 |
| AT - $H_2O$ + PtCl | 1508.6084 | 754.3036 | 84.8 | 2 | 2.80 |
| a6 | 756.4157 | 756.4151 | 71.6 | 1 | 2.29 |
| b8y8 | 757.4037 | 757.4032 | 88.5 | 1 | 2.48 |
| AT + PtCl | 1526.6190 | 763.3089 | 90.3 | 2 | 2.81 |
| y6 | 763.4255 | 763.4250 | 89.0 | 1 | 2.53 |
| b6 - $NH_3$ | 767.3840 | 767.3835 | 87.7 | 1 | 2.21 |
| b6 | 784.4106 | 784.4100 | 89.3 | 1 | 2.33 |
| b9y7 | 795.3942 | 795.3937 | 90.9 | 1 | 2.26 |
| b5 + Pt | 840.3008 | 840.3014 | 57.5 | 1 | 1.14 |
| AT + Pt($C_{13}H_{14}O_4$) | 1724.7315 | 862.3652 | 88.83 | 2 | 2.97 |
| a9y8 | 866.4677 | 866.4672 | 83.1 | 1 | 1.96 |
| b9y8 | 894.4626 | 894.4621 | 87.7 | 1 | 2.08 |
| y7 | 926.4888 | 926.4883 | 91.0 | 1 | 2.28 |

| | | | | | |
|---|---|---|---|---|---|
| a6 - $H_2O$ +Pt | 933.3699 | 933.3694 | 75.3 | 1 | 2.67 |
| a6 + Pt | 949.3648 | 949.3654 | 88.5 | 1 | 1.36 |
| b6 + Pt | 977.3597 | 977.3603 | 89.6 | 1 | 1.52 |
| a8 | 1000.5369 | 1000.5363 | 89.1 | 1 | 1.92 |
| y8 | 1025.5573 | 1025.5567 | 93.2 | 1 | 2.07 |

## Substance P

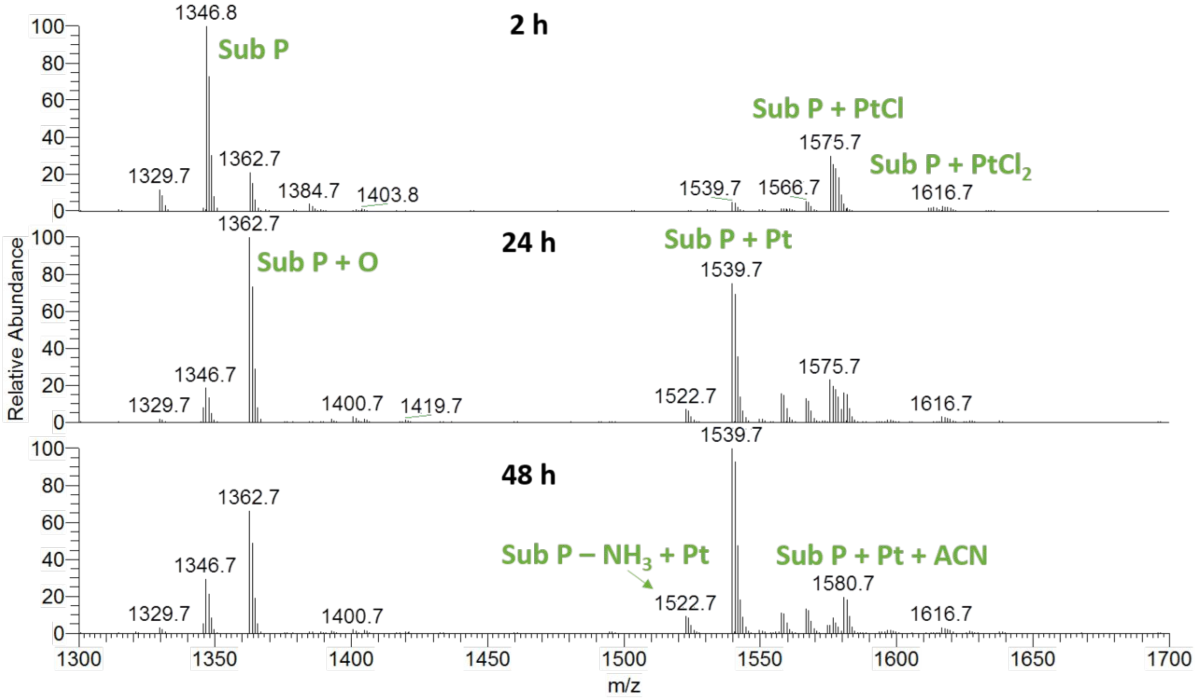


*Figure S1: Deconvoluted mass spectra of Sub P incubated with an equimolar amount of ASA-buten-$PtCl_3$.*

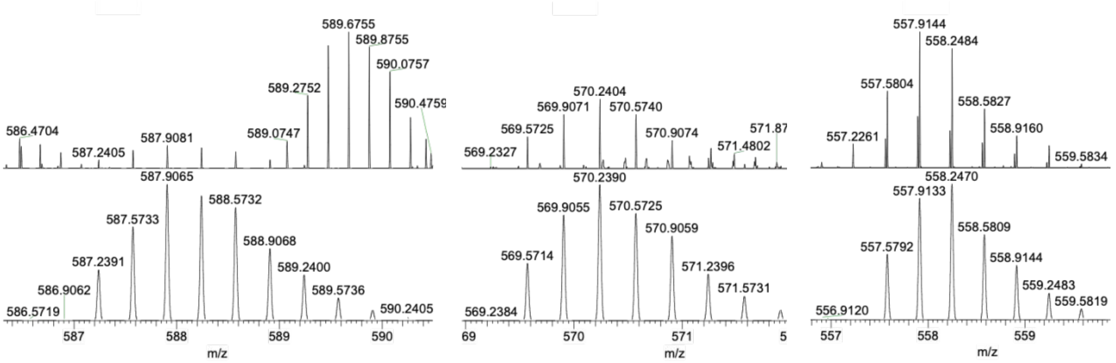


*Figure S2: Experimental (top) and calculated (bottom) isotopic distributions for [Sub P + $PtCl_2$ + y1 + 4H]$^{3+}$ (left), [Sub P – $NH_3$ + PtCl + y1 + 3H]$^{3+}$ (middle) and [Sub P – $NH_3$ + Pt +y1 + 2H]$^{3+}$ (right).*

*Table S2: Full list of fragments obtained from HCD fragmentation at 30% NCE of the precursor m/z 593.1 (z =5) (Sub P)$_2$ + $PtCl_2$.*

| fragment | $m_{calc.}$ | $m_{exp.}$ | Similarity | Charge | error [ppm] |
|---|---|---|---|---|---|
| Phe | 120.0813 | 120.0808 | 97.4 | 1 | 1.33 |
| Gln | 129.0664 | 129.0659 | 88.2 | 1 | 1.53 |
| b1 - $NH_3$ | 140.0824 | 140.0818 | 92.4 | 1 | 1.78 |

| | | | | | |
|---|---|---|---|---|---|
| a10y3 | 143.1184 | 143.1179 | 93.2 | 1 | 1.51 |
| y1 | 149.0749 | 149.0743 | 80.7 | 1 | 1.52 |
| b1 | 157.1089 | 157.1084 | 80.6 | 1 | 1.49 |
| b10y3 | 171.1134 | 171.1128 | 89.5 | 1 | 1.46 |
| a9y4 | 177.1028 | 177.1022 | 84.5 | 1 | 1.59 |
| a5y8 | 198.1243 | 198.1237 | 90.9 | 1 | 1.76 |
| b9y4 | 205.0977 | 205.0972 | 87.2 | 1 | 1.74 |
| b5y8 | 209.0926 | 209.0921 | 86.9 | 1 | 1.91 |
| b5y8 | 226.1192 | 226.1186 | 90.1 | 1 | 1.92 |
| b3y10 | 226.1556 | 226.1550 | 91.5 | 1 | 1.90 |
| b4y9 | 226.1556 | 226.1550 | 91.5 | 1 | 1.90 |
| a7y6 - $NH_3$ | 231.1134 | 231.1128 | 91.4 | 1 | 1.68 |
| b2 - $NH_3$ | 237.1352 | 237.1346 | 81.4 | 1 | 1.84 |
| b6y7 - $NH_3$ | 240.0984 | 240.0979 | 87.2 | 1 | 2.03 |
| b4 | 480.3173 | 240.1581 | 85.5 | 2 | 2.05 |
| b2 | 254.1617 | 254.1612 | 92.2 | 1 | 1.93 |
| a8y5 | 267.1497 | 267.1492 | 91.7 | 1 | 1.74 |
| b7y6 | 276.1348 | 276.1343 | 87.7 | 1 | 1.60 |
| a5 - $NH_3$ | 563.3544 | 281.6766 | 81.9 | 2 | 1.75 |
| a10y4 | 290.1869 | 290.1863 | 82.4 | 1 | 1.67 |
| b5 - $NH_3$ | 591.3493 | 295.6741 | 93.2 | 2 | 1.72 |
| b5 | 608.3758 | 304.1874 | 88.3 | 2 | 1.82 |
| b10y4 | 318.1818 | 318.1812 | 87.3 | 1 | 1.48 |
| b4y10 | 323.2083 | 323.2078 | 85.5 | 1 | 1.57 |
| a6y8 | 326.1828 | 326.1823 | 85.4 | 1 | 1.82 |
| a8 | 1003.5841 | 334.5275 | 90.6 | 3 | 1.95 |
| b6 - 2 $NH_3$ | 702.3813 | 351.1901 | 89.7 | 2 | 1.64 |
| b6y8 | 354.1777 | 354.1772 | 92.0 | 1 | 1.74 |
| b6 - $NH_3$ | 719.4079 | 359.7034 | 87.6 | 2 | 1.85 |
| b6 | 736.4344 | 368.2167 | 88.6 | 2 | 1.50 |
| b3 | 382.2567 | 382.2561 | 93.7 | 1 | 1.75 |
| b10 | 1201.6846 | 400.5610 | 87.7 | 3 | 2.07 |
| b7y7 | 404.1934 | 404.1928 | 79.6 | 1 | 1.65 |
| a7 - 2 $NH_3$ | 821.4548 | 410.7269 | 87.8 | 2 | 1.90 |
| a7 - NH3 | 838.4814 | 419.2401 | 92.8 | 2 | 1.76 |
| b8y6 | 423.2032 | 423.2027 | 81.4 | 1 | 2.02 |
| a7 | 855.5079 | 427.7534 | 92.5 | 2 | 1.72 |
| a10y5 | 437.2553 | 437.2547 | 77.5 | 1 | 1.77 |
| b7 | 883.5028 | 441.7509 | 92.9 | 2 | 1.80 |
| b9y6 - $NH_3$ | 463.1981 | 463.1976 | 87.8 | 1 | 1.82 |
| a7y8 | 473.2512 | 473.2507 | 88.2 | 1 | 1.74 |
| a8 - $NH_3$ | 985.5498 | 492.7743 | 86.2 | 2 | 2.04 |
| b7y8 | 501.2462 | 501.2456 | 87.3 | 1 | 1.71 |
| a8 | 1002.5763 | 501.2876 | 90.1 | 2 | 1.93 |
| b8 - $NH_3$ | 1013.5447 | 506.7718 | 92.7 | 2 | 1.84 |
| Sub P - $NH_3$ + Pt | 1525.6742 | 508.5575 | 89.4 | 3 | 1.94 |
| Sub P + Pt | 1542.7008 | 514.2330 | 71.9 | 3 | 1.96 |
| b8 | 1030.5712 | 515.2851 | 92.0 | 2 | 1.67 |
| Sub P - $NH_3$ + PtCl | 1561.6509 | 520.5497 | 90.8 | 3 | 2.02 |
| a9 - $NH_3$ | 1042.5712 | 521.2851 | 94.9 | 2 | 1.69 |
| b9 - 2 $NH_3$ | 1053.5396 | 526.7693 | 90.9 | 2 | 1.72 |
| a9 | 1059.5978 | 529.7983 | 91.7 | 2 | 1.86 |
| b9 - $NH_3$ | 1070.5661 | 535.2825 | 92.8 | 2 | 1.80 |

| | | | | | |
|---|---|---|---|---|---|
| b9 | 1087.5927 | 543.7958 | 94.0 | 2 | 1.71 |
| a10 - 2 $NH_3$ | 1138.6287 | 569.3138 | 92.9 | 2 | 2.09 |
| a10 - NH3 | 1155.6553 | 577.8271 | 94.9 | 2 | 2.04 |
| a5 | 579.3731 | 579.3725 | 86.7 | 1 | 1.90 |
| a10 | 1172.6818 | 586.3404 | 93.9 | 2 | 1.65 |
| b10 - $NH_3$ | 1183.6502 | 591.8246 | 92.5 | 2 | 1.79 |
| b10 | 1200.6768 | 600.3378 | 95.0 | 2 | 1.72 |
| b5 | 607.3680 | 607.3675 | 91.7 | 1 | 2.07 |
| b7y9 - $NH_3$ | 612.3146 | 612.3140 | 85.7 | 1 | 1.14 |
| b8y8 | 648.3146 | 648.3140 | 81.5 | 1 | 2.10 |
| Sub P - $NH_3$ | 1331.7172 | 665.8581 | 92.1 | 2 | 2.12 |
| Sub P | 1348.7438 | 674.3713 | 92.4 | 2 | 2.00 |
| a6 | 707.4317 | 707.4311 | 85.1 | 1 | 2.21 |
| b6 - $NH_3$ | 718.4000 | 718.3995 | 84.2 | 1 | 2.28 |
| b6 | 735.4266 | 735.4260 | 92.6 | 1 | 2.33 |
| Sub P - $NH_3$ + Pt | 1524.6664 | 762.3326 | 92.3 | 2 | 2.37 |
| Sub P + Pt | 1541.6929 | 770.8459 | 90.0 | 2 | 2.66 |
| Sub P - $NH_3$ + PtCl | 1560.6431 | 780.3210 | 92.5 | 2 | 2.46 |
| Sub P + PtCl | 1577.6696 | 788.8343 | 88.1 | 2 | 2.56 |
| b10y8 | 818.4201 | 818.4196 | 80.0 | 1 | 2.15 |
| b9y9 | 833.4310 | 833.4304 | 88.9 | 1 | 2.37 |
| a7 | 854.5001 | 854.4995 | 88.8 | 1 | 2.19 |
| b7 - $NH_3$ | 865.4684 | 865.4679 | 94.7 | 1 | 2.05 |
| b7 | 882.4950 | 882.4944 | 92.1 | 1 | 2.13 |
| a10y9 | 918.5201 | 918.5196 | 83.4 | 1 | 1.77 |
| b10y9 - $NH_3$ | 929.4885 | 929.4880 | 87.6 | 1 | 1.63 |
| b10y9 | 946.5151 | 946.5145 | 92.0 | 1 | 1.99 |
| a8 - $NH_3$ | 984.5419 | 984.5414 | 92.3 | 1 | 1.52 |
| a8 | 1001.5685 | 1001.5679 | 90.1 | 1 | 1.90 |
| b8 | 1029.5634 | 1029.5629 | 92.6 | 1 | 2.13 |
| a9 | 1058.5900 | 1058.5894 | 90.6 | 1 | 2.14 |
| b9 - $NH_3$ | 1069.5583 | 1069.5578 | 87.2 | 1 | 1.75 |
| b9 | 1086.5849 | 1086.5843 | 93.8 | 1 | 1.98 |
| a10 - $NH_3$ | 1154.6475 | 1154.6469 | 87.8 | 1 | 0.99 |
| a10 | 1171.6740 | 1171.6735 | 91.3 | 1 | 1.67 |
| b10 - $NH_3$ | 1182.6424 | 1182.6418 | 84.3 | 1 | 1.48 |
| b10 | 1199.6689 | 1199.6684 | 92.0 | 1 | 1.73 |

## Ubiquitin

*Table S3: Adducts of UQ and ASA-buten-$PtCl_3$.*

| Adduct | $m_{exp.}$ | $m_{calc}$ | error [ppm] |
|---|---|---|---|
| UQ | 8564.6462 | 8564.6302 | 1.87 |
| UQ + O (Met oxidation) | 8580.6441 | 8580.6250 | 2.23 |
| Ac-UQ | 8606.6569 | 8606.6406 | 1.89 |
| $Ac_2$-UQ | 8648.6681 | 8648.6509 | 1.99 |
| UQ + Pt | 8757.5965 | 8757.5785 | 2.06 |
| UQ + PtCl | 8793.5735 | 8793.5531 | 2.32 |

| | | | |
|---|---|---|---|
| Ac-UQ + Pt | 8799.6148 | 8799.5890 | 2.93 |
| UQ + $PtCl_2$ | 8830.5665 | 8830.5300 | 4.13 |
| $Ac_2$-UQ + Pt | 8841.6393 | 8841.5994 | 4.51 |
| UQ + $PtCl_2$ + ACN | 8871.5788 | 8871.5574 | 2.41 |
| UQ + Pt($C_{13}H_{14}O_4$) | 8991.6881 | 8991.6678 | 2.26 |
| UQ + Pt($C_{13}H_{14}O_4$)($H_2O$) | 9009.6940 | 9009.6780 | 1.78 |
| UQ + PtCl($C_{13}H_{14}O_4$) | 9027.6848 | 9027.6428 | 4.65 |
| Ac-UQ + Pt($C_{13}H_{14}O_4$) | 9033.6990 | 9033.6779 | 2.34 |
| Ac-UQ + Pt($C_{13}H_{14}O_4$)($H_2O$) | 9051.7056 | 9051.6888 | 1.86 |
| UQ + $PtCl_2$($C_{13}H_{14}O_4$) | 9064.6687 | 9064.6199 | 5.38 |

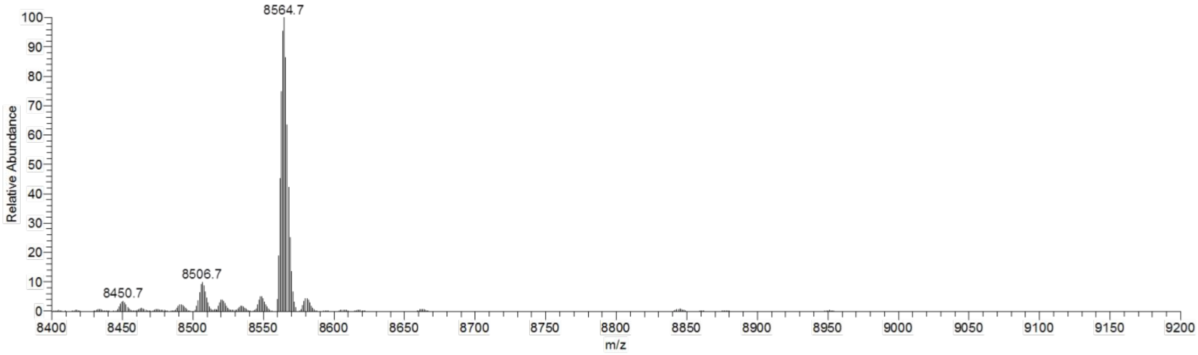


*Figure S3: Deconvoluted mass spectrum of UQ that has been incubated with 5 eq. ASA for 1 week.*

*Table S4: Full list of fragments obtained from HCD fragmentation at 25% NCE of the precursor m/z 901.9 (z =10) UQ + Pt($C_{13}H_{14}O_4$)($H_2O$) after 2 h.*

| fragment | $m_{calc.}$ | $m_{exp.}$ | Similarity | Charge | error [ppm] |
|---|---|---|---|---|---|
| **Thr** | 102.0555 | 102.0550 | 95.0 | 1 | -0.64 |
| **Glu - CO** | 102.0555 | 102.0550 | 95.0 | 1 | -0.64 |
| **His - CO** | 110.0718 | 110.0713 | 70.2 | 1 | -0.30 |
| **Phe - CO** | 120.0813 | 120.0808 | 90.6 | 1 | -0.18 |
| **Lys** | 129.1028 | 129.1022 | 74.9 | 1 | 0.38 |
| **Glu** | 130.0504 | 130.0499 | 92.5 | 1 | 0.53 |
| **Tyr - CO** | 136.0762 | 136.0757 | 93.1 | 1 | 0.42 |
| **a36y42** | 143.1184 | 143.1179 | 91.7 | 1 | 0.49 |
| **a20y58** | 157.0977 | 157.0972 | 88.9 | 1 | 0.17 |
| **a38y40** | 167.1184 | 167.1179 | 93.5 | 1 | 0.26 |
| **a39y39** | 185.0926 | 185.0921 | 91.2 | 1 | 0.19 |
| **b20y58** | 185.0926 | 185.0921 | 91.2 | 1 | 0.19 |
| **Gly-Lys** | 186.1243 | 186.1237 | 91.7 | 1 | 0.21 |
| **a26y52** | 186.1243 | 186.1237 | 91.7 | 1 | 0.21 |
| **Thr-Leu(Ile) - CO** | 187.1447 | 187.1441 | 91.3 | 1 | 0.18 |
| **b38y40** | 195.1134 | 195.1128 | 90.7 | 1 | 0.37 |
| **a19y59** | 199.1083 | 199.1077 | 94.1 | 1 | 0.36 |
| **a61y17** | 200.1399 | 200.1394 | 75.3 | 1 | 0.64 |
| **Ala-Lys** | 200.1399 | 200.1394 | 75.3 | 1 | 0.64 |

| | | | | | |
|---|---|---|---|---|---|
| **b57y21** | 201.1239 | 201.1234 | 82.0 | 1 | 0.30 |
| **Val-Glu - CO** | 201.1239 | 201.1234 | 82.0 | 1 | 0.30 |
| **Ser-Asp** | 203.0668 | 203.0662 | 81.1 | 1 | 0.72 |
| **Gln-Leu(Ile) - CO** | 214.1556 | 214.1550 | 89.7 | 1 | 0.46 |
| **Thr-Leu(Ile)** | 215.1396 | 215.1390 | 91.2 | 1 | 0.19 |
| **Leu(Ile)-Glu - CO** | 215.1396 | 215.1390 | 91.2 | 1 | 0.19 |
| **b65y13** | 217.0824 | 217.0819 | 92.6 | 1 | 0.48 |
| **a52y26** | 217.0824 | 217.0819 | 92.6 | 1 | 0.48 |
| **b22y56** | 217.0824 | 217.0819 | 92.6 | 1 | 0.48 |
| **a5y73** | 219.1497 | 219.1492 | 83.1 | 1 | 0.72 |
| **b19y59** | 227.1032 | 227.1026 | 84.6 | 1 | 0.76 |
| **b61y17** | 228.1348 | 228.1343 | 80.6 | 1 | 0.61 |
| **Val-Lys** | 228.1712 | 228.1707 | 89.5 | 1 | 0.53 |
| **Val-Glu** | 229.1188 | 229.1183 | 86.2 | 1 | 0.20 |
| **Gln-Leu(Ile)** | 242.1505 | 242.1499 | 91.3 | 1 | 0.73 |
| **Leu(Ile)-Glu** | 243.1345 | 243.1339 | 91.5 | 1 | 0.36 |
| **Asp-Gln** | 244.0933 | 244.0928 | 92.3 | 1 | 0.65 |
| **b25y53** | 244.0933 | 244.0928 | 92.3 | 1 | 0.65 |
| **b33y45** | 244.1297 | 244.1292 | 85.7 | 1 | 0.78 |
| **Gln-Lys** | 257.1614 | 257.1608 | 88.6 | 1 | 0.66 |
| **b48y31** | 257.1614 | 257.1608 | 88.6 | 1 | 0.66 |
| **b2** | 260.1069 | 260.1063 | 80.8 | 1 | 0.62 |
| **Ile-Phe** | 261.1603 | 261.1598 | 84.4 | 1 | 0.45 |
| **b60y18** | 278.1141 | 278.1135 | 85.0 | 1 | 1.03 |
| **a35y44** | 287.1719 | 287.1714 | 82.6 | 1 | 0.49 |
| **b12y67** | 287.1719 | 287.1714 | 82.6 | 1 | 0.49 |
| **b11y68** | 287.1719 | 287.1714 | 82.6 | 1 | 0.49 |
| **a19y60** | 298.1767 | 298.1761 | 84.3 | 1 | 0.81 |
| **b21y58** | 300.1196 | 300.1190 | 91.7 | 1 | 0.52 |
| **b36y43** | 300.1559 | 300.1554 | 82.2 | 1 | 0.88 |
| **b22y57** | 304.1145 | 304.1139 | 91.4 | 1 | 0.50 |
| **b39y40** | 310.1403 | 310.1397 | 88.6 | 1 | 0.76 |
| **a24y55** | 316.1872 | 316.1867 | 89.7 | 1 | 0.48 |
| **a16y63** | 316.1872 | 316.1867 | 89.7 | 1 | 0.48 |
| **b14y65** | 316.1872 | 316.1867 | 89.7 | 1 | 0.48 |
| **b9y70** | 316.1872 | 316.1867 | 89.7 | 1 | 0.48 |
| **b19y60** | 326.1716 | 326.1710 | 82.2 | 1 | 1.02 |
| **b29y50** | 328.2349 | 328.2343 | 81.4 | 1 | 1.08 |
| **b17y62** | 342.2029 | 342.2023 | 81.5 | 1 | 0.78 |
| **b26y53** | 343.1618 | 343.1612 | 80.9 | 1 | 1.18 |
| **b24y55** | 344.1822 | 344.1816 | 90.0 | 1 | 0.56 |
| **b16y63** | 344.1822 | 344.1816 | 90.0 | 1 | 0.56 |
| **b32y47** | 357.1774 | 357.1769 | 85.4 | 1 | 0.78 |
| **b25y54** | 357.1774 | 357.1769 | 85.4 | 1 | 0.78 |
| **b52y27** | 358.1614 | 358.1609 | 84.5 | 1 | 1.01 |
| **b18y61** | 358.1614 | 358.1609 | 84.5 | 1 | 1.01 |

| | | | | | |
|---|---|---|---|---|---|
| **b34y45** | 373.1723 | 373.1718 | 86.1 | 1 | 0.97 |
| **a22y58** | 373.1723 | 373.1718 | 86.1 | 1 | 0.97 |
| **b3** | 373.1910 | 373.1904 | 80.2 | 1 | 0.94 |
| **y7** | 771.5079 | 385.7534 | 84.0 | 2 | 1.12 |
| **b47y33** | 389.2189 | 389.2183 | 84.9 | 1 | 0.89 |
| **b4y75** | 389.2189 | 389.2183 | 84.9 | 1 | 0.89 |
| **a36y44** | 400.2560 | 400.2554 | 81.3 | 1 | 0.90 |
| **b13y67** | 400.2560 | 400.2554 | 81.3 | 1 | 0.90 |
| **b11y69** | 400.2560 | 400.2554 | 81.3 | 1 | 0.90 |
| **b22y58** | 401.1672 | 401.1667 | 92.4 | 1 | 0.71 |
| **a21y59** | 401.1672 | 401.1667 | 92.4 | 1 | 0.71 |
| **y4** | 402.2465 | 402.2459 | 88.8 | 1 | 0.82 |
| **b43y40** | 836.4505 | 418.2247 | 87.5 | 2 | 0.95 |
| **b42y41** | 836.4505 | 418.2247 | 87.5 | 2 | 0.95 |
| **b16y68** | 845.4858 | 422.7424 | 79.0 | 2 | 1.40 |
| **b36y44** | 428.2509 | 428.2504 | 86.1 | 1 | 0.81 |
| **a26y54** | 428.2509 | 428.2504 | 86.1 | 1 | 0.81 |
| **a16y64** | 429.2713 | 429.2708 | 83.3 | 1 | 1.36 |
| **b15y65** | 429.2713 | 429.2708 | 83.3 | 1 | 1.36 |
| **b30y50** | 441.3189 | 441.3184 | 78.9 | 1 | 0.78 |
| **y8** | 884.5920 | 442.2954 | 87.2 | 2 | 1.09 |
| **a18y62** | 443.2506 | 443.2500 | 84.0 | 1 | 1.13 |
| **b17y63** | 443.2506 | 443.2500 | 84.0 | 1 | 1.13 |
| **b14y66** | 444.2822 | 444.2817 | 93.4 | 1 | 1.11 |
| **b9y71** | 444.2822 | 444.2817 | 93.4 | 1 | 1.11 |
| **b2 + Pt** | 455.0717 | 455.0711 | 75.9 | 1 | 1.06 |
| **b16y64** | 457.2662 | 457.2657 | 69.6 | 1 | 1.03 |
| **a52y28** | 458.2251 | 458.2245 | 86.3 | 1 | 1.01 |
| **b25y55** | 458.2251 | 458.2245 | 86.3 | 1 | 1.01 |
| **b24y56** | 459.2091 | 459.2086 | 87.6 | 1 | 1.12 |
| **b18y62** | 471.2455 | 471.2449 | 87.6 | 1 | 1.07 |
| **b44y40** | 949.5345 | 474.7667 | 86.8 | 2 | 0.88 |
| **b43y41** | 949.5345 | 474.7667 | 86.8 | 2 | 0.88 |
| **y13** | 1452.8651 | 484.2878 | 89.2 | 3 | 1.60 |
| **a23y58** | 486.2564 | 486.2558 | 85.6 | 1 | 1.08 |
| **b14y67** | 501.3037 | 501.3031 | 84.5 | 1 | 1.14 |
| **b13y68** | 501.3037 | 501.3031 | 84.5 | 1 | 1.14 |
| **b12y69** | 501.3037 | 501.3031 | 84.5 | 1 | 1.14 |
| **b11y70** | 501.3037 | 501.3031 | 84.5 | 1 | 1.14 |
| **b10y71** | 501.3037 | 501.3031 | 84.5 | 1 | 1.14 |
| **y9** | 1021.6509 | 510.8249 | 90.5 | 2 | 1.04 |
| **b23y58** | 514.2513 | 514.2508 | 85.7 | 1 | 1.06 |
| **a20y61** | 514.2513 | 514.2508 | 85.7 | 1 | 1.06 |
| **a36y45** | 515.2829 | 515.2824 | 73.9 | 1 | 1.07 |
| **b4** | 520.2594 | 520.2588 | 73.5 | 1 | 1.53 |
| **y14** | 1580.9600 | 526.9861 | 92.9 | 3 | 1.15 |

| | | | | | |
|---|---|---|---|---|---|
| **a3 + Pt** | 540.1608 | 540.1603 | 86.0 | 1 | 1.27 |
| **a19y62** | 540.3033 | 540.3028 | 69.7 | 1 | 0.90 |
| **b36y45** | 543.2779 | 543.2773 | 84.4 | 1 | 1.18 |
| **b45y40** | 1096.6029 | 548.3009 | 81.0 | 2 | 1.41 |
| **y10** | 1134.7349 | 567.3669 | 87.2 | 2 | 1.27 |
| **b3 + Pt** | 568.1557 | 568.1552 | 81.9 | 1 | 1.66 |
| **y15** | 1709.0186 | 569.6723 | 91.8 | 3 | 1.43 |
| **b18y63** | 572.2932 | 572.2926 | 82.8 | 1 | 1.44 |
| **b34y52** | 1155.6612 | 577.8300 | 85.9 | 2 | 1.39 |
| **b33y53** | 1155.6612 | 577.8300 | 85.9 | 2 | 1.39 |
| **b46y40** | 1167.6400 | 583.8195 | 84.5 | 2 | 1.43 |
| **b27y54** | 584.3408 | 584.3402 | 65.2 | 1 | 1.43 |
| **y16** | 1822.1027 | 607.3670 | 92.5 | 3 | 1.19 |
| **y11** | 1235.7826 | 617.8908 | 93.0 | 2 | 1.19 |
| **b5** | 619.3278 | 619.3272 | 72.1 | 1 | 1.45 |
| **y17** | 1936.1456 | 645.3813 | 90.0 | 3 | 1.30 |
| **y12** | 1322.8147 | 661.4068 | 92.9 | 2 | 1.01 |
| **b16 + Pt** | 1999.9895 | 666.6626 | 86.7 | 3 | 2.21 |
| **a4 + Pt** | 685.2136 | 685.2141 | 80.4 | -1 | 0.08 |
| **b17 + Pt** | 2099.0579 | 699.6854 | 84.4 | 3 | 1.58 |
| **y18** | 2099.2089 | 699.7358 | 93.7 | 3 | 1.15 |
| **b15 + Pt($C_{13}H_{14}O_4$)** | 2105.0361 | 701.6781 | 85.6 | 3 | 1.87 |
| **y37** | 4258.3662 | 709.7272 | 93.6 | 6 | 1.25 |
| **y25** | 2844.5570 | 711.1387 | 93.9 | 4 | 1.45 |
| **b4 + Pt** | 713.2085 | 713.2091 | 85.7 | -1 | -0.13 |
| **b15y68** | 715.4354 | 715.4349 | 72.9 | 1 | 1.45 |
| **b14y69** | 715.4354 | 715.4349 | 72.9 | 1 | 1.45 |
| **b13y70** | 715.4354 | 715.4349 | 72.9 | 1 | 1.45 |
| **y13** | 1451.8572 | 725.9281 | 94.0 | 2 | 1.05 |
| **y58** | 6536.5593 | 726.2838 | 85.8 | 9 | 0.49 |
| **y19** | 2214.2359 | 738.0781 | 89.7 | 3 | 1.62 |
| **b18 + Pt** | 2228.1005 | 742.6996 | 87.8 | 3 | 2.15 |
| **y26** | 2973.5996 | 743.3993 | 92.8 | 4 | 1.74 |
| **b16y67** | 743.4303 | 743.4298 | 80.5 | 1 | 1.63 |
| **b16 + Pt($C_{13}H_{14}O_4$)** | 2234.0787 | 744.6923 | 90.3 | 3 | 1.72 |
| **b25y58** | 757.3368 | 757.3363 | 82.0 | 1 | 1.54 |
| **y40** | 4567.4987 | 761.2492 | 93.9 | 6 | 1.53 |
| **a17 + Pt($c_{13}H_{14}O_4$)** | 2305.1522 | 768.3835 | 81.1 | 3 | 1.54 |
| **y27** | 3086.6836 | 771.6704 | 94.5 | 4 | 1.39 |
| **b17 + Pt($C_{13}H_{14}O_4$)** | 2333.1471 | 777.7151 | 91.5 | 3 | 2.03 |
| **b32y51** | 783.4729 | 783.4723 | 80.4 | 1 | 1.34 |
| **b30y53** | 783.4729 | 783.4723 | 80.4 | 1 | 1.34 |
| **b29y54** | 783.4729 | 783.4723 | 80.4 | 1 | 1.34 |
| **y14** | 1579.9522 | 789.9756 | 94.3 | 2 | 1.02 |
| **y49** | 5551.0513 | 793.0068 | 78.9 | 7 | 1.55 |
| **y28** | 3214.7422 | 803.6850 | 93.8 | 4 | 1.50 |

| | | | | | |
|---|---|---|---|---|---|
| **y21** | 2414.3520 | 804.7834 | 65.7 | 3 | 0.08 |
| **y43** | 4866.6468 | 811.1073 | 70.0 | 6 | 1.66 |
| **y50** | 5679.1462 | 811.3061 | 82.5 | 7 | 1.48 |
| **b33y50** | 812.4994 | 812.4989 | 71.7 | 1 | 1.32 |
| **y58** | 6535.5515 | 816.9434 | 94.8 | 8 | 1.21 |
| **b18 + Pt($C_{13}H_{14}O_4$)** | 2462.1897 | 820.7293 | 93.0 | 3 | 1.96 |
| **y51** | 5778.2146 | 825.4587 | 83.0 | 7 | 1.31 |
| **y44** | 4994.7418 | 832.4564 | 93.3 | 6 | 1.17 |
| **y59** | 6664.5941 | 833.0737 | 88.2 | 8 | 0.67 |
| **y29** | 3342.8372 | 835.7087 | 82.2 | 4 | 1.49 |
| **y22** | 2515.3996 | 838.4660 | 76.9 | 3 | 1.54 |
| **b15** | 1676.9899 | 838.4944 | 82.5 | 2 | 1.68 |
| **b38** | 4197.3096 | 839.4614 | 65.7 | 5 | 2.33 |
| **y52** | 5892.2576 | 841.7505 | 92.9 | 7 | 1.18 |
| **b16y68** | 844.4780 | 844.4775 | 68.6 | 1 | 1.56 |
| **y30** | 3399.8586 | 849.9641 | 83.9 | 4 | 3.03 |
| **y37** | 4257.3584 | 851.4711 | 95.0 | 5 | 1.22 |
| **y45** | 5109.7687 | 851.6276 | 91.0 | 6 | 0.42 |
| **y15** | 1708.0108 | 854.0048 | 95.0 | 2 | 0.93 |
| **b26y58** | 856.4052 | 856.4047 | 81.8 | 1 | 1.19 |
| **y53** | 6021.3002 | 860.1852 | 92.5 | 7 | 0.68 |
| **b75 + Pt** | 8687.6121 | 868.7607 | 64.6 | 10 | 0.65 |
| **y62** | 7005.7891 | 875.7231 | 77.8 | 8 | 1.07 |
| **UQ + Pt** | 8762.6441 | 876.2639 | 89.9 | 10 | 1.54 |
| **a74 + Pt($C_{13}H_{14}O_4$)** | 8836.6849 | 883.6679 | 64.3 | 10 | 2.83 |
| **b75** | 8921.7013 | 892.1696 | 65.3 | 10 | 2.77 |
| **UQ + Pt($C_{13}H_{14}O_4$)** | 8996.7333 | 899.6728 | 87.2 | 10 | 0.51 |
| **y56** | 6350.4588 | 907.2079 | 94.6 | 7 | 1.32 |
| **y24** | 2728.5222 | 909.5069 | 86.1 | 3 | 0.98 |
| **y16** | 1821.0949 | 910.5469 | 84.7 | 2 | 0.77 |
| **y73** | 8196.5040 | 910.7221 | 80.9 | 9 | 1.69 |
| **y40** | 4566.4909 | 913.2976 | 95.6 | 5 | 0.97 |
| **y57** | 6437.4909 | 919.6410 | 91.5 | 7 | 0.96 |
| **y49** | 5550.0434 | 925.0067 | 83.9 | 6 | 1.18 |
| **y58** | 6534.5436 | 933.5057 | 95.7 | 7 | 1.05 |
| **y50** | 5678.1384 | 946.3559 | 93.2 | 6 | 1.90 |
| **y59** | 6663.5862 | 951.9403 | 94.3 | 7 | 1.05 |
| **y69** | 7720.2167 | 965.0265 | 90.2 | 8 | 4.01 |
| **y60** | 6762.6546 | 966.0930 | 94.3 | 7 | 1.40 |
| **y43** | 4865.6390 | 973.1272 | 93.1 | 5 | 1.61 |
| **y70** | 7821.2644 | 977.6575 | 92.9 | 8 | 2.72 |
| **y61** | 6891.6972 | 984.5276 | 76.1 | 7 | 0.61 |
| **y26** | 2972.5918 | 990.8634 | 81.2 | 3 | 1.29 |
| **y44** | 4993.7340 | 998.7462 | 94.5 | 5 | 0.97 |
| **b16 + Pt** | 2000.9973 | 1000.4981 | 65.5 | 2 | 0.60 |
| **y62** | 7004.7813 | 1000.6825 | 92.8 | 7 | 1.11 |

| | | | | | |
|---|---|---|---|---|---|
| **y53** | 6020.2923 | 1003.3815 | 94.1 | 6 | 1.25 |
| **b52 + Pt** | 6033.1141 | 1005.5185 | 88.9 | 6 | 0.85 |
| **y63** | 7105.8290 | 1015.1179 | 91.5 | 7 | 1.39 |
| **y54** | 6133.3764 | 1022.2289 | 92.7 | 6 | 1.81 |
| **y73** | 8195.4962 | 1024.4365 | 82.1 | 8 | 0.87 |
| **y27** | 3085.6758 | 1028.5581 | 88.0 | 3 | 1.07 |
| **y55** | 6234.4241 | 1039.0701 | 91.3 | 6 | 1.83 |
| **y46** | 5236.8195 | 1047.3633 | 85.3 | 5 | 1.98 |
| **y18** | 2098.2011 | 1049.1000 | 92.4 | 2 | 0.98 |
| **y56** | 6349.4510 | 1058.2413 | 94.6 | 6 | 0.69 |
| **y37** | 4256.3506 | 1064.0871 | 95.4 | 4 | 0.85 |
| **y47** | 5349.9035 | 1069.9802 | 77.8 | 5 | 1.19 |
| **y28** | 3213.7344 | 1071.2443 | 83.7 | 3 | 1.10 |
| **y57** | 6436.4830 | 1072.7466 | 91.0 | 6 | 0.38 |
| **y58** | 6533.5358 | 1088.9221 | 95.7 | 6 | 0.71 |
| **y59** | 6662.5784 | 1110.4292 | 91.0 | 6 | 0.36 |
| **y39** | 4468.4303 | 1117.1070 | 96.7 | 4 | 1.10 |
| **y60** | 6761.6468 | 1126.9406 | 91.7 | 6 | 0.80 |
| **y50** | 5677.1306 | 1135.4256 | 89.1 | 5 | 1.19 |
| **y40** | 4565.4830 | 1141.3702 | 96.7 | 4 | 0.61 |
| **y42** | 4735.5886 | 1183.8966 | 94.9 | 4 | 0.74 |
| **y53** | 6019.2845 | 1203.8564 | 92.1 | 5 | 0.37 |
| **y43** | 4864.6312 | 1216.1572 | 95.5 | 4 | 0.61 |
| **y54** | 6132.3686 | 1226.4732 | 87.4 | 5 | 0.42 |
| **y44** | 4992.7261 | 1248.1810 | 90.4 | 4 | 0.66 |
| **y58** | 6532.5280 | 1306.5050 | 92.6 | 5 | 2.31 |
| **y46** | 5235.8116 | 1308.9524 | 88.0 | 4 | 1.92 |
